# Conjunctival Autograft versus Combined Amniotic Membrane and Mini-Simple Limbal Epithelial Transplant for Primary Pterygium Excision

**DOI:** 10.18502/jovr.v17i1.10164

**Published:** 2022-01-21

**Authors:** Ashok Jha, Abhay Simba

**Affiliations:** ^1^Department of Ophthalmology, Military Hospital Gaya, Gaya, India; ^2^Department of Ophthalmology, Anugrah Narayan Medical College, Gaya, India

**Keywords:** Conjunctival Autograft (CAG), Mini-simple Limbal Epithelial Transplant (mini-SLET), Pterygium

## Abstract

**Purpose:**

To compare outcomes of conjunctival autograft (CAG) and combined amniotic membrane with mini-simple limbal epithelial transplant (mini-SLET) after primary pterygium excision.

**Methods:**

All consenting adults with primary pterygium were included in this study. After pterygium excision, patients were randomized to receive either CAG or mini-SLET and both grafts were held in place with fibrin glue. The patients were followed-up at days 1, 3, 7, 14, and 30 and subsequently at the third, sixth, and ninth months. The recurrence rate was considered as the primary outcome measure whereas the operating time, postoperative symptoms, and surgical complications were considered the secondary outcome measures.

**Results:**

The study comprised of 264 eyes of 264 patients, of which 233 (88%) completed the nine months of follow-up. Of these, 118 (51%) received CAG and 115 (49%) received mini-SLET. The groups were comparable at baseline. Recurrence of pterygium was seen in two (1.6%) eyes in the CAG group and three (2.6%) eyes in the mini-SLET group (*P* = 0.68). Operative time for mini-SLET (20.33 ± 1.28 min) was significantly higher than that for CAG (12.01 ± 1.26 min) (*P* < 0.001). Graft displacement was observed in one case in group II (*P *= 0.999). The Lim Bon Siong (foreign body sensation, lacrimation, pain, and irritation) score in the CAG group was statistically significant for all four symptoms at days 1 and 3; however, at day 7, foreign body sensation, pain, and irritation scores were significantly higher for the CAG group.

**Conclusion:**

In this study, the overall recurrence rate was very low and comparable between mini-SLET and the established technique of CAG after performing the primary pterygium excision. Despite a longer surgical time, mini-SLET appears to be a viable option for the management of primary pterygium.

##  INTRODUCTION

Pterygium is a benign, fleshy triangular ocular lesion that can cross the limbus and encroach on the cornea, with subsequent visual impairment.^[1]^ It most often involves nasal conjunctiva and may necessitate surgical removal if associated with visual impairment, astigmatic refractive errors, or cosmetic concerns. Postoperative recurrence is not uncommon, hence surgical excision has been coupled with various adjunctive measures like beta irradiation^[2]^ and anti-metabolites such as Mitomycin C.^[3,4]^ Although these methods are relatively safe, complications such as secondary bacterial infection, punctate keratitis, scleral melting, and raised intraocular pressure (IOP) have been reported. To prevent these side effects and achieve superior results in terms of graft stability and potentially lower recurrence rates, human amniotic membrane grafting (AMG)^[5]^ and conjunctival autografting (CAG)^[6]^ were introduced to cover the bare sclera after pterygium excision. Of these, ipsilateral CAG is now the surgical procedure of choice, owing to the ease of the procedure and the difficulties in procuring AMG. Additionally, the efficacy and low recurrence rate of the CAG method has been corroborated by many authors.^[6,7,8,9]^

Tissue adhesives like fibrin glue^[10]^ which are used to secure the grafts in place after pterygium excision present many benefits such as lesser operating time, reduced discomfort during the postoperative period, and reduced complications associated with sutures.^[11,12]^ Alternatively, the CAG can be secured using autologous in situ blood coagulum.^[13,14,15]^

More recently, Hernández-Bogantes et al have described a technique of using a combination of AMG and small pieces of autologous limbal epithelial cells (mini-simple limbal epithelial transplant [SLET])^[16]^ to cover the bare sclera using tissue adhesive. Authors reported excellent outcomes with this new technique, albeit in only 10 eyes. Given these encouraging results, we believe that this may be an alternative technique to CAG for surgical management of pterygium. At present there are very few head to head studies comparing outcomes of the well-established CAG with the relatively new mini-SLET after primary pterygium excision. Hence, this study was performed to assess the efficacy and recurrence rate after the two aforementioned procedures.

##  METHODS 

This prospective, randomized, interventional study was duly approved by the local Institutional Ethics Committee (Ethical clearance certificate no. 29/MH/2015 dated Aug 11, 2015). This study was conducted in accordance with the tenets of the Declaration of Helsinki. Patient enrollment occurred between August 2015 and January 2019.

All consecutive adult patients visiting the outpatient department of our hospital with primary pterygium and requiring surgical excision for cosmesis, intense foreign body sensation, and reduced vision either due to induced astigmatism or encroachment on the visual axis were invited to participate in the study. Patients who agreed to follow-up for nine months after surgery were recruited after their informed consent. Patients with other ocular surface disorders, hypersensitivity to blood constituents, and seropositivity to Hepatitis B, Hepatitis C, and HIV were excluded from the study.

Sample size calculation was based on presumed differences in the recurrence rates of pterygium in the two groups. Given 1:1 randomization, 90% power, and a precision error of 5% to detect a difference of 10% or more in proportion of patients experiencing recurrent pterygium, a required minimum sample size of 230 eyes (115 in each group) was obtained. To account for a 15% loss to follow-up, we recruited 264 patients for the study.

Using simple randomization, the patients were divided into two groups: one received the CAG and the other received the mini-SLET treatment. This simple randomization utilized serially generated computer codes along with allocation concealment. A parallel allocation strategy was used in a 1:1 allocation. The evaluating ophthalmologist (AS) was masked to the type of graft used. The operating surgeon (AJ) and patients were masked to the procedural details and the type of graft used. The sealed envelopes for the type of graft were opened just before the completion of the pterygium excision. The graft status was masked in all cases at every follow-up visit during the clinical testing.

**Figure 1:**
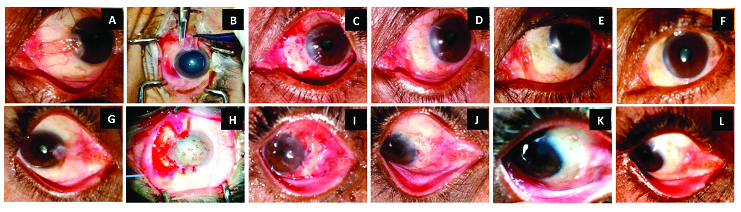
Serial clinical photographs of two patients of primary pterygium: (Patient 1 [A ∼ F]; Patient 2 [G ∼ L]): preoperative, intraoperative, and postoperative. Pterygium excision with CAG with fibrin glue (A–F) and pterygium excision with mini-SLET (G–L). (A) A case of primary nasal pterygium (left eye[LE]) before pterygium excision with CAG. (B) Dissection of pterygium. (C) POD 1. (D) POD 30 graft well taken. (E & F) Postoperative months 6 and 9, respectively: restoration of near normal anatomy without any recurrence. (G) A case of primary nasal pterygium (Right eye [RE]) before pterygium excision with mini-SLET. (H) Intraoperative photograph showing dissected pterygium with eight limbal pieces. (I) POD 1. (J) POD 30: well-settled graft. (K & L) Postoperative months 6 and 9, respectively: restoration of near normal anatomy without any signs of recurrence.

Thorough medical and ocular history and demographic details such as age and gender of the participants were obtained. Thereafter, a comprehensive examination of the eyes included best-corrected visual acuity assessment, slit-lamp evaluation of the pterygium, and the anterior and posterior segment evaluation using a +90D lens. Pterygia were divided into three grades based on the classification proposed by Tan and coworkers:^[17]^ T1 = unobscured and distinguished episcleral vessels underneath the body of the pterygium; T2 = partially obscured or indistinct episcleral vessels; and T3 = completely obscured episcleral vessels by fibrovascular tissue. Serial clinical photographs of patients were taken preoperatively, per-operatively, and postoperatively on days 1 and 30 in addition to the sixth and ninth months for both cohorts [Figure 1].

Peribulbar anesthesia (2% lidocaine hydrochloride) was used for all of the surgeries, which were performed by a single surgeon (AJ). Westcott scissors were used to draw horizontal incisions along the superior and inferior borders of the body of pterygium. Subsequently, using Moorefield's conjunctival forceps, the pterygium was reflected toward the limbus making another peripheral incision parallel to the limbus. The remaining fibrovascular tissues underneath the bulbar conjunctiva were dissected and excised to the maximum possible extent.

The bare sclera was then measured by a caliper. In the CAG group, a near tenon-free CAG, which was 1 mm larger in area than the bare sclera was harvested from the supero-temporal region. This thin graft was stuck to the exposed sclera using fibrin glue (Baxter, TISSEEL) with correct orientation. The angled flat part of two iris spatulas was maneuvered horizontally and vertically utilized to expand the graft to its maximum possible size besides removing excess glue from the sclera bed.

In the mini-SLET cohort, after an initial one clock hour (10–11 o'clock for right eye and 1–2 o'clock for left eye) peritomy performed with Westcott scissors, a limbal tissue 2 × 2 mm in size was excised with the help of a crescent blade. Using Vannas scissors, this strip was then sliced into six to eight pieces under increased magnification. These pieces were then affixed on the inlay AMG closer to the limbus using fibrin glue. Thereafter, an AMG overlay was used to maintain graft pieces in the exact position. Freeze-dried AMG (Amnio-care, Biocover Labs, India), available in 3 × 3 cm size was used for the study. A bandage contact lens was placed on the cornea at the end of the procedure.

Using a patented fibrinotherm device, the fibrin glue (TISSEEL VH, Baxter AG) used in the above described procedures was prepared by reconstituting thrombin and freeze-dried protein concentrate in calcium chloride and fibrinolysis inhibitor solutions, respectively. Both thrombin and fibrin were suctioned into separate syringes, which were mounted with a 27G canula. To use the glue, almost an equal number of drops of fibrin and thrombin were utilized for both the cohorts. Total operating time was noted for both the groups. Mitomycin C was not used in either group.

In the first week of the postoperative period, patients received 1% prednisolone acetate and 0.5% moxifloxacin eye drops every 4 and 6 hr, respectively, followed by tapering dosages of topical steroid, which continued for one month. The patients were reviewed on postoperative days 1, 3, 7, 14, and subsequently at months 1, 3, 6, and 9.

While early postoperative visits involved the assessment of graft positioning, later visits included reporting of the recurrence and disintegration of limbal pieces on the slit lamp by a masked investigator. Recurrence was considered the primary outcome measure, which was defined as any fibrovascular growth that occurred at the surgical site at any time during the follow-up period. Operating time and surgical complications were considered the secondary outcome measures. Four symptoms (foreign body sensation, epiphora [watering], pain and irritation) were evaluated, based on a 5-point scale adapted from Lim Bon Siong R et al. The patients were asked to complete a questionnaire in which 0 meant no pain, 1 meant presence of easily tolerable pain, 2 meant pain causing some discomfort, 3 meant presence of pain partially interfering with sleep or usual activity, and 4 meant pain completely interfering with usual activity or sleep.

### Statistical Analysis 

The statistical analysis was performed using SPSS (Statistical Package for the Social Sciences) software version 20. A *P*-value < 0.05 was considered statistically significant. Comparison of continuous variables such as age, preoperative BCVA, operative time, and the dimensions of graft between the two groups was done using the parametric unpaired *t*-test. Pearson's Chi-square test or the Fisher's exact test were used for comparative analysis of categorical variables like gender, laterality, grades of pterygium, and indication of surgery between the two groups.

##  RESULTS

A total of 264 consecutive eyes of 264 patients were enrolled in the study, of which 31 eyes (15 in CAG and 16 in mini-SLET groups) were ruled out due to inadequate follow-up. Hence, data from 233 eyes (*n* = 118 CAG and 115 in mini-SLET) that finished nine months of follow-up was included in the final analysis. Table 1 depicts the baseline characteristics of CAG and mini-SLET groups. Baseline characteristics were statistically insignificant between the two groups. The comparative analysis of graft size and the operating time has been summarized in Table 2. The mean operative time for mini-SLET group (20.33 ± 1.28 min) was significantly higher (*P* < 0.001) than the CAG group (12.01 ± 1.26 min). Four postoperative symptoms have been compared in Table 3 using the nonparametric Mann–Whitney U-test. Both groups exhibited foreign body sensation until day 7. A statistically significant difference in the median score of foreign body sensation was noted on day 1 (*P* < 0.001), day 3 (*P* < 0.001), and day 7 (*P *< 0.001) between the CAG and mini-SLET groups. This symptom improved significantly between days 1 and 7 in both group I (*P* < 0.001) and group II (*P* < 0.001) as demonstrated by the Wilcoxon signed-rank test. Both groups showed persistence of epiphora (watering) till day 7. A statistically significant difference in the median score of epiphora (watering) was observed on day 1 (*P* < 0.001) and day 3 (*P* < 0.001) between the two groups. It was noted that this symptom, too, showed significant improvement at days 1 and 7 in both group I (*P* < 0.001) and group II (*P* < 0.001). Pain persisted until days 7 and 3 in groups I and II, respectively. Both the study and control groups exhibited significant difference in the median score of pain on day 1 (*P* < 0.001), day 3 (*P* <0.001), and day 7 (*P* = 0.008). Despite a median score of 6 and 0, the significant difference at days 1 and day 7, respectively, is ascribed to the variance in the distribution of pain score between the two groups. A significant improvement in this symptom was observed between day 1 and day 7 in both the groups (*P* < 0.001). Irritation in the operated eye persisted until day 7 in both the groups. The median score for irritation was noted to be statistically significant on day 1 (*P* < 0.001), day 3 (*P *< 0.001), and day 7 (*P* < 0.001) between the two groups. Both the study and control groups showed significant improvement in this symptom between day 1 and day 7 (*P* < 0.001), and between day 1 and day 14, respectively.

Recurrence was observed at the first, third, sixth, and ninth months. Two (1.6%) eyes in the CAG group exhibited recurrence whereas three (2.6%) had recurrence in mini-SLET group (*P* = 0.681). Two patients in the CAG group and one in the mini-SLET group showed recurrence within three months. Recurrence in the remaining two patients in the mini-SLET group was observed between the third and sixth months. Two cases in each group underwent revision surgery whereas one patient in the mini-SLET group refused surgery. Within the six months follow-up period, no recurrence was observed in any of the patients who underwent revision surgery.

One eye (0.87%) in the mini-SLET group exhibited AMG displacement on the first postoperative day, which was repositioned on the very same day. None of the eyes in the study experienced any other adverse effects related to the grafts.

**Table 1:** Comparison of baseline characteristics between the CAG Group and the mini-SLET group


**Characteristics**	**CAG group (** ***n*** ** = 118) **	**Mini-SLET group (** ***n*** ** = 115)**	*P* **-value**
Age (yr)	53.81 ± 14.28 (range = 22–80)	52.38 ± 14.62 (range = 26–78)	0.446 ${}^{*}$
Sex	F = 49.15% (*n* = 58)	F = 44.35% (*n* = 51)	0.437 ${}^{†}$
	M = 50.85% (*n* = 60)	M = 55.65% (*n* = 64)	
Laterality	Right eye = 57.6% (*n* = 68)	Right eye = 53.9% (*n* = 62 )	0.696 ${}^{†}$
	Left eye = 42.4% (*n* = 50)	Left eye = 46.1% (*n* = 53)	
Grade		
I	13.5% (*n* = 16)	12.2% (*n* = 14)	0.920 ${}^{†}$
II	61.9% (*n* = 73)	61.7% (*n* = 71)	
III	24.6% (*n* = 29)	26.1% (*n* = 30)	
Occupation	Outdoor = 61.1% (*n* = 72)	Outdoor = 67.8% (*n* = 78)	0.282 ${}^{†}$
	Indoor = 38.9% (*n* = 46)	Indoor = 32.2% (*n* = 37)	
Indications for surgery		0.895 ${}^{†}$
Cosmesis	40.7% (*n* = 48)	40% (*n* = 46)	
Foreign body sensation	29.7% (*n* = 35)	26.1% (*n *= 30)	
Reduced VA due to Astigmatism	16.1% (*n* = 19)	22.6% (*n* = 26)	
Threatening Visual Axis	13.5% (*n* = 16)	11.3% (*n *= 13)	
Preoperative BCVA (LogMar)	0.46 ± 0.38 (range = 0–1.46)	0.43 ± 0.26 (range = 0.16–1.18)	0.476 ${}^{*}$
${}^{*}$ Unpaired *t*-test; ${}^{†}$ χ ${}^{2}$ test			

**Table 2:** Comparison of the size of the graft and operative time between the CAG group and mini-SLET group


**Measures**	**CAG group (** ***n*** ** = 118) **	**Mini-SLET group (** ***n*** ** = 115)**	*P* **-value**
Dimensions of the graft (mm)		
Horizontal	5.10 ± 0.41 (range = 4.2–6.5)	5.14 ± 0.45 (range = 4.5–6.8)	0.448 ${}^{*}$
Vertical	6 ± 0.32 (range = 5.5–8.0)	6.09 ± 0.54 (range = 5.5–8.0)	0.097 ${}^{*}$
Operative time (min)	12.01 ± 1.26 (range = 10.1–14.0)	20.33 ± 1.28 (range = 18–22)	<0.001 ${}^{*}$
${}^{*}$ Unpaired *t*-test			

**Table 3:** Comparative outcome of the postoperative symptom score between the CAG group and mini-SLET group


	**CAG group (** ***n*** ** = 118) **			**Mini-SLET group (** ***n*** ** = 115)**			*P* -value ${}^{*}$
	**Min**	**Max**	**Median**	**Min**	**Max**	**Median**	
Foreign body sensation				
Day 1	3	9	9	0	9	6	**<0.001**
Day 3	0	9	6	0	6	3	**<0.001**
Day 7	0	6	3	0	3	0	**<0.001**
Day 14	0	6	0	0	0	0	0.255
Day 30	0	0	0	0	0	0	0.999
Epiphora (watering)				
Day 1	0	9	6	0	9	6	**<0.001**
Day 3	0	6	3	0	6	3	**<0.001**
Day 7	0	6	0	0	6	0	0.511
Day 14	0	0	0	0	0	0	0.999
Day 30	0	0	0	0	0	0	0.999
Pain				
Day 1	0	9	6	0	6	3	**<0.001**
Day 3	0	9	3	0	6	0	**<0.001**
Day 7	0	6	0	0	6	0	**0.008**
Day 14	0	6	0	0	0	0	0.089
Day 30	0	0	0	0	0	0	0.999
Irritation				
Day 1	3	9	9	0	9	6	**<0.001**
Day 3	0	9	6	0	6	3	**<0.001**
Day 7	0	6	3	0	3	0	**<0.001**
Day 14	0	6	0	0	6	0	0.183
Day 30	0	0	0	0	0	0	0.999
Statistically significant values are in boldface after ${}^{*}$ Mann–Whitney U-test																																																																																			

##  DISCUSSION

In this randomized study, we found a very low rate of recurrence in eyes that received CAG versus mini-SLET, with statistically insignificant difference in the recurrence rates between the two groups. The operating time was significantly higher in the mini-SLET group, however, none of the eyes experienced intra- or postoperative complications attributable to the surgery.

Pterygium is postulated to occur due to localized dysfunction of nasal limbal stem cells consequential to exposure to UVB light.^[18]^ This theory forms the basis of the incorporation of limbal stem cells in the surgical treatment of pterygium. Of all the techniques described in literature,^[3,4][5][6]^ CAG modality has been found to be associated with the lowest recurrence rate.^[7,8,10]^ The CAG can be attached to the bare sclera by sutures, fibrin glue, or autologous in situ blood coagulum. Amongst the three techniques of fixing CAG, fibrin glue definitely scores over others with the least operative time.^[19]^

Use of SLET for the treatment of limbal stem cell deficiency was first advocated by Sangwan et al.^[20]^ Subsequently, mini-SLET^[16]^ was innovatively used for treating 10 patients suffering with primary pterygium. The basis of using mini-SLET for pterygium surgery was localized deficiency or dysfunction of limbal stem cells.^[21,22]^ The AMG acts as a basement membrane and a substrate supporting the growth of epithelial progenitor cells.^[23]^ AMG is also endowed with anti-inflammatory properties owing to the presence of protease inhibitors.^[24]^ However, AMG itself is devoid of limbal stem cells, hence its solitary use to cover the bare sclera after pterygium excision, despite providing a mechanical barrier, does not address the underlying pathology of limbal stem cell deficiency. As a result, this may in turn lead to potentially more recurrences and tilt the balance toward CAG in terms of beneficial outcomes.

An extensive MEDLINE search did not reveal any similar study of this magnitude; hence we undertook a randomized trial to compare the aforementioned two techniques as mini-SLET appeared to be a viable prospect for the treatment of pterygium.^[16]^ In 2015, Hernández-Bogantes E et al,^[16]^ elicited this interesting innovation in 10 patients with primary pterygium without any recurrence after eight months of follow-up. Sati et al^[25]^ reported a randomized control trial, comparing the outcomes between 42 cases of CAG and 40 cases of mini-SLET. The study reported a 9.5% recurrence in the CAG group and 2.5% recurrence in the mini-SLET group. Although clinically meaningful, these differences were not found to be statistically significant. We experienced much lower rates of recurrence in the CAG group and almost similar in the mini-SLET group. Mini-SLET incorporates the use of AMG and limbal stem cells, which could lower recurrence rates as is evident in our study. Mini-SLET is a miniaturized modification of the original SLET technique first described by Sangwan et al.^[20]^ However, mini-SLET technique scores over SLET in terms of placing the limbal stem cells' pieces over amniotic membrane in proximity to the limbus. In our opinion, mini-SLET offers an alternative solution to patients with insufficient conjunctiva due to prior surgeries or those suspected of suffering with glaucoma. Additionally, it is more likely to restore the normal anatomy of the limbus.

There were a few limitations of the study such as the relatively low overall numbers of recurrences, which made it difficult to make robust conclusions while using statistical tools. Follow-up of < 12 months and the considerable number of excluded eyes (12%) also contributed toward limiting our evaluations. The strength of this study included its randomized and masked study design, the relatively large sample size, and the longer follow-up period, which exceeded that of other similar studies.

In summary, the overall recurrence rate was very low in this study and comparable between mini-SLET and the established technique of CAG for primary pterygium excision. Despite a longer surgical time, mini-SLET appears to be a viable alternative to CAG in the management of primary pterygium. The mini-SLET may be considered as the procedure of choice for all cases of primary pterygium surgery, especially, if cost and availability are not an issue. Also, mini-SLET is more likely to replace the abnormal limbal stem cells compared to CAG. In particular, this should be adopted as the procedure of choice as conjunctival-sparing surgery (especially in young patients, glaucoma, and cicatrizing conjunctivitis).

##  Financial Support and Sponsorship

The authors did not receive any financial support for the research, authorship, and/or publication of this article.

##  Conflicts of Interest

The authors report no conflict of interest.
